# Factors in Gestational Diabetes Mellitus Predicting the Needs for Insulin Therapy

**DOI:** 10.1155/2016/4858976

**Published:** 2016-07-11

**Authors:** Ya Zhang, Jiashen Shao, Feifei Li, Xianming Xu

**Affiliations:** Department of Obstetrics and Gynecology, Shanghai General Hospital, Shanghai Jiao Tong University, No. 100 Haining Road, Shanghai 200080, China

## Abstract

*Objective.* To identify factors predicting the need for insulin therapy in pregnancies complicated by gestational diabetes mellitus (GDM).* Methods*. A total of 1352 patients with GDM diagnosed by the 75-g/2-h oral glucose tolerance test (OGTT) were enrolled in this study. Univariate and multivariate analysis were performed; receiver operating characteristics (ROC) were also drawn.* Results*. There was a significant difference in factors such as maternal age, pregestational BMI, first visit SBP, first visit DBP, FBG of first visit, FBG at time of OGTT, 75-g OGTT glucose value (fasting, after 1 h and 2 h), and serum HbA1c level at diagnosis between patients with insulin therapy and patients with medical nutrition therapy (MNT) alone. Multivariate analysis showed that higher FBG at time of OGTT, first 75 g OGTT 2 h plasma glucose, and HbA1c concentration at diagnosis lead to more likely need of insulin therapy.* Conclusion*. The probability of insulin therapy can be estimated in pregnant women with GDM based on fasting and 2 h glucose values during OGTT and HbA1c value at diagnosis of GDM.

## 1. Introduction

Gestational diabetes mellitus (GDM) is a carbohydrate metabolism disorder with onset or first recognition as early as during pregnancy [1]. GDM is significantly associated with maternal hypertensive disorders and increased rate of cesarean delivery, as well as perinatal complications. Studies have demonstrated that treatment of GDM reduces serious perinatal morbidity [2, 3]. The majority of studies of GDM have shown that self-glucose monitoring is very useful to maintain glycemic control in GDM and can improve pregnancy outcomes. Controlling blood glucose levels is the primary goal in managing women with GDM. Medical nutrition therapy (MNT) and exercise are the mainstay of treatment for GDM, but if this fails, antenatal insulin treatment (AIT) is needed to reduce glucose levels in order to ensure normal fetal development and better perinatal outcomes [2]. Thus, the need for insulin therapy might be a characteristic for patients with more severe GDM due to a greater difficulty in glycemic control [4]. Studies have demonstrated that some factors are underlying the requirement of insulin therapy for glycemic control in pregnancies complicated by GDM, such as diagnosis of GDM at an early gestational age, obesity, family history of diabetes, exacerbated fetal growth, fasting glucose levels, the values of the abnormal blood glucose level of 75 g oral glucose tolerance tests (OGTT), and the value of glycated hemoglobin [2].

The aim of this study is to compare the characteristics of women who required insulin therapy with those who needed MNT only during their pregnancy complicated by GDM and to identify the factors predicting insulin need or leading to an increased need for insulin therapy in these patients. These predictive factors were then used to estimate the threshold of the risks for insulin needs in GDM.

## 2. Methods

The study was approved by the Ethics Committee of Shanghai General Hospital, Shanghai Jiao Tong University. Since the data were collected retrospectively, no informed consent form was signed by any patient.

1352 pregnant women diagnosed with GDM by OGTT at Shanghai First People's Hospital (Shanghai's GAM Center) between January 2009 and December 2012 were enrolled in this study. All patients underwent the 75-g, 2-h oral glucose tolerance test (75-g 2-h OGTT) between 24 and 34 weeks of gestation. Before the OGTT, all the women had a diet with no carbohydrate restriction in 3 days. Samples were collected from antecubital veins just before and at first and second hours after 300 mL oral glucose solution intake containing 75 g glucose after 12 hours of fasting. The diagnosis of GDM was established when a patient presented one or more 75-g OGTT values equal to or above the reference values recommended by the International Association of Diabetes and Pregnancy Study Groups (IADPSG) 2010 guidelines: fasting <5.1 mmol/L; 1 h <10 mmol/L; 2 h <8.5 mmol/L [5]. The inclusion criteria are as follows: (1) diagnosis with GDM by OGTT; (2) voluntary participation in the study; (3) no previous insulin treatment; and (4) no diabetic complications. The exclusion criteria are as follows: (1) patient (Pt) was treated with insulin previously and (2) Pt has diabetic complications.

All pregnant women with a diagnosis of GDM were systematically evaluated and received medical nutrition therapy (MNT) from a nutritionist. The diet was divided into seven servings, with 35 kcal kg-ideal-weight−1 day−1 or 25 kcal kg-current-weight−1 day−1 (in cases of obese patients), and 300 kcal/day was added in the second and third trimester of gestation. Physical activity for 30 min per day was recommended to all patients. Patients were educated in regard of self-monitoring of finger sticks daily. According to Fifth International Workshop Conference on GDM [3], the target maternal capillary glucose levels were <5.3 mmol/L at fasting and <7.8 mmol/L at 1 h and <6.7 mmol/L at 2 h after having the meal. Women who failed to achieve target glucose levels after two weeks of MNT need insulin therapy.

### 2.1. Statistical Analysis

Univariate analysis was performed to compare clinical variables and characteristics of patients with AIT and those with MNT only. Means of the continuous variables were compared by Student's *t*-test and the frequencies variables in the two groups were compared by Pearson's *χ* ^2^-test. Then a multivariate stepwise logistic regression was preformed; *P* value of independent variables entered or removed in model was defined as 0.1. *Z*-test was used for comparison on area under curve (AUC) of receiver operating characteristic (ROC). Values of *P* < 0.05 were considered statistically significant. All statistical analyses were performed using SAS 9.1.

## 3. Results

Data were collected from 1352 women with GDM between January 2009 and December 2012 at Shanghai First People's Hospital. Of these women, 44.8% required AIT during their pregnancy while the rest were maintained on MNT alone.  Table 1 shows the comparison of clinical variables and characteristic between patients with AIT and those with MNT only. Factors such as maternal age, pregestational BMI, first visit SBP, first visit DBP, FBG of first visit, FBG at time of OGTT, 75-g OGTT glucose value (1 h and 2 h during the test), and serum HbA1c level at diagnosis exhibited a significant difference between the two groups (*P* < 0.05). No significant difference was seen in gestational age of first visit, gestational age of OGTT, and prior pregnancy history between the two groups (*P* > 0.05).

We also performed a multivariate stepwise logistic regression with all factors in Table 1 as independent variable, with need of insulin therapy as the dependent variable. As an optimization of logistic regression model, independent variables including pregestational BMI, FBG at time of OGTT, first 75 g OGTT 2 h plasma glucose, and HbA1c concentration at diagnosis were retained in the model. The results of stepwise logistic regression analysis are shown in Table 2. The ORs of one unit increasing on pregestational BMI, FBG at time of OGTT, first 75 g OGTT 2 h plasma glucose, and HbA1c level at diagnosis for need of insulin therapy were 1.08 (95% CI, 1.03–1.13), 2.02 (95% CI, 1.65–2.47), 1.90 (95% CI, 1.46–2.47), and 1.16 (95% CI, 1.07–1.27), respectively.

**Figure 1:**
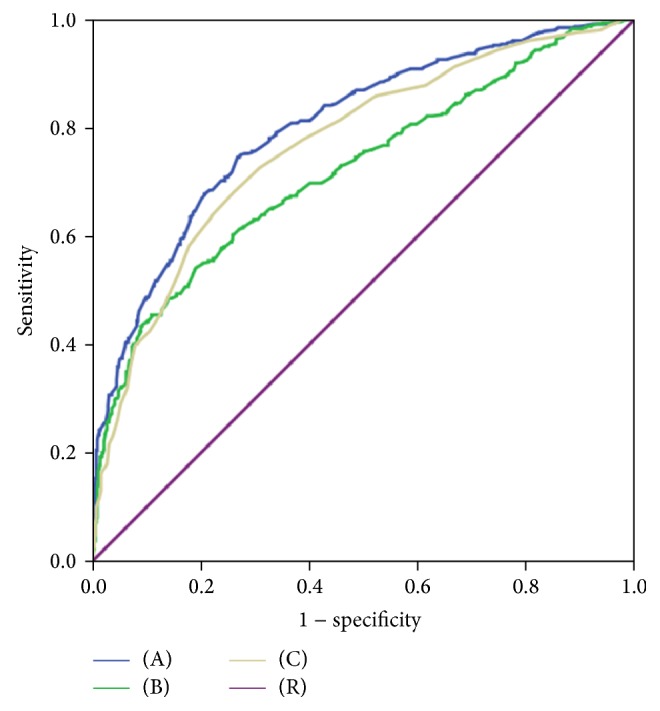
Receiver operating characteristic (ROC) curve of FBG, HbA1c, and OGTT 2 h plasma glucose for need of AIT. (A) FBG at time of OGTT; (B) OGTT 2 h plasma glucose; (C) HbA1c at time of OGTT; (R) reference line.

Area under curve (AUC) of receiver operating characteristic (ROC) was generated to determine diagnostic value of pregestational BMI, FBG at time of OGTT, first 75 g OGTT 2 h plasma glucose, and HbA1c level at diagnosis (data were showed in Table 3). In regard to FBG at time of OGTT, the AUC of ROC was 0.80 (95% CI, 0.78–0.82), the cutoff value was 5.69 mmol/L, sensitivity and specificity were 0.673 and 0.797, respectively, and the Youden index of cutoff value was 0.47. In regard to HbA1c at time of OGTT, the AUC of ROC was 0.77 (95% CI, 0.74–0.80), the cutoff value was 5.85%, sensitivity and specificity were 0.693 and 0.732, respectively, and the Youden index of cutoff value was 0.425. In regard to the OGTT 2 h plasma glucose, the AUC of ROC was 0.73 (95% CI, 0.70–0.76), the cutoff value was 10.8 mmol/L, sensitivity and specificity were 0.612 and 0.754, respectively, and the Youden index of cutoff value was 0.366. In regard to pregestational BMI, AUC of ROC was 0.508 and *P* > 0.05 when compared with referenced line (AUC = 0.5) indicated lack of diagnostic values. Diagnostic value of FBG at time of OGTT for need of insulin therapy was better than first 75 g OGTT 2 h plasma glucose and HbA1c concentration at diagnosis (*P* < 0.05).  Figure 1 showed the ROC of FBG at time of OGTT, 75 g OGTT 2 h plasma glucose, and HbA1c level at diagnosis.

## 4. Discussion

It is well known that gestational diabetes mellitus (GDM) is a carbohydrate metabolism disorder. Self-monitoring of blood glucose is very effective in maintaining glycemic control in GDM [6]. Some experts showed that self-monitoring of blood glucose is efficacious both in identifying women who will require insulin therapy for management and in adjusting therapy once insulin is started [7]. In addition to self-glucose monitoring, identification of factors associated with the need for insulin therapy is of clinical importance in the care planning process. In this study, we identified that maternal age, family history of diabetes, obesity, prior GDM, 75-g OGTT glucose control (fasting and 1 h and 2 h during the test), and serum HbA1c concentrations were all significantly different between women with GDM who required insulin therapy and those who could be controlled on MNT alone. Moreover, we found that fasting glucose level, the 2 h glucose level during OGTT, and serum HbA1c concentrations at diagnosis were predictors for insulin use among women with GDM. Fasting glucose level ≥5.69 mmol/L, 2 h glucose level ≥10.8 mmol/L during OGTT, or HbA1c level ≥5.85% at diagnosis was found to distinguish the cases that required insulin in addition to MNT.

In our study, 44.8% women required AIT during their pregnancy with GDM. It is significantly higher compared with that reported in the ACHOIS study, where only 20% of women required AIT [2]. The most important reason for the difference we think is ethnic difference. First, it has been reported that GDM mothers were more likely to be overweight/obese and of Asian ethnicity [8]. Second, in a recent study trying to determine the factors that were associated with suboptimal glucose control in type II diabetes, Asian ethnicity was found to be one of the contributing factors [9]. Third, in terms of GDM, it was also found that the prevalence of GDM was also the highest in Asian ethnicity, and although it was very difficult to compare the proportion of GDM patients that need insulin therapy across from different regions, limited data did support that there were great variations among different ethnicities; for example, women with GDM from southeast Asia had the lowest prevalence of insulin therapy (37.2%), while Anglo-Europeans has the highest (56.7%) [10, 11]. Based on the above facts, though there are no definitive studies regarding the insulin therapy in GDM with Chinese ethnicity, it will not be surprising if it is found to be different from that of the other ethnicities published in the literature which is exactly what we found in this study.

Besides ethnicity, differences in diagnostic standard and target goals for fasting glucose control can also contribute to the higher proportion of AIT in our study compared with that of ACHOIS. For example, our study requires a target fasting blood glucose level to be below 5.3, while ACHOIS target level was 5.6.

Our study showed that elevated fasting glucose level on OGTT is a strong predictor for insulin therapy, and this has been consistent with previous studies [2]. Langer [12] found significant positive association between fasting glucose >105 mg/dL (5.83 mmol/L) and maternal and perinatal complications such as fetal macrosomia, neonatal hypoglycemia, hypertensive syndromes, and cesarean delivery and concluded that fasting glucose levels above this value are indicative of need for insulin therapy. In addition, the author reported that 70% of pregnant women with fasting glucose ≤95 mg/dL (5.27 mmol/L) achieve satisfactory glycemic control with diet programs, whereas only 30% of women with fasting glucose >95 mg/dL (5.27 mmol/L) achieve adequate glucose levels with nutritional therapy. Another study [13] had suggested that a fasting glucose level of 87 mg/dL (4.83 mmol/L) can accurately predict the need for insulin with a sensitivity of 89.1% when compared with the 1 or 2 h values on 75 g OGTT during pregnancy. More recently, Bakiner et al. [14] found a significant relationship between fasting glucose levels ≥89.5 mg/dL (4.97 mmol/L) and the need for insulin treatment in gestational diabetes. Of course, different cutoff values may be related to methodology of studies, different standards of diagnosing GDM, various target glucose levels, or ethnic differences.

Recently, the importance of the 2-h glucose level during OGTT in predicting the needs for insulin therapy is less clear. Our study shows that there is a significant difference between AIT and MNT groups on univariate analysis, and the 2-h glucose level during OGTT is an independent predictor of AIT in the multivariate model. However, other studies had suggested that elevation of the 3-h rather than the 2-h glucose level during OGTT has greater predictive value for AIT during pregnancy [2].

Obesity has been associated with increasing insulin resistance and impaired beta cell function [2]. Previous study [15] showed that, compared with normal weight and overweight women, obese women achieved satisfactory metabolic control only when they were treated with insulin. And obese women had a 2- to 3-fold higher risk of perinatal complications while they achieved metabolic control only with diet. It shows that obesity is an important factor for the indication of insulin therapy. In our study, obesity was not independent predictor for insulin therapy, even though it exhibited a significant difference between AIT and MNT groups.

Glycated hemoglobin (HbA1c) was found to be useful for the prediction of insulin requirement. González-Quintero et al. [16] found that HbA1c ≥ 6% at diagnosis of GDM was an independent risk factor for insulin requirement later in pregnancy. Sapienza et al. [17] also showed that HbA1c is the independent predictor for insulin need. More recently, Bakiner et al. [14] found that HbA1c is independent predictor for AIT, and the threshold level of ≥5.485% in HbA1c at diagnosis was found to distinguish the cases that required insulin in addition to MNT.

The limitations of our study include those inherent in a retrospective study. First, the retrospective design of our study is a limitation for controlling the dietary compliance of the participants. In addition, unmeasured fetal or placental factors that influence insulin resistance may also have big impacts on antenatal insulin treatment. Therefore, the positive predictive value of fasting glucose and 2 h glucose levels of OGTT and HbA1c level at diagnosis is relatively low; they are not specific predictors for insulin treatment in patients with GDM. Finally, the time window of OGTT testing in this study was 24–34 weeks, while the guidelines suggest testing between 24 and 28 weeks, but this should not have compromised this study because the majority of the data were still collected within the time period defined by the guidelines.

## 5. Conclusion

Our study confirmed that women with GDM who required insulin therapy differ greatly in many ways from those who can be managed on MNT only. Recognizing patient characteristics that predict failure of MNT at an early stage after diagnosis of GDM may assist the diabetes team in prioritizing care and resource allocation in order to serve patient's medical interests better.

## Figures and Tables

**Table 1:** Clinical variables and characteristics of patients with AIT and those with MNT only.

Variable	AIT	MNT	*P* ^*∗*^
	*n* = 606	*n* = 746	
Age, mean ± SD (years)	31.44 ± 5.29	30.85 ± 4.89	<0.001
Pregestational BMI, mean ± SD (kg/m^2^)	24.62 ± 3.86	22.77 ± 3.55	<0.001
First visit SBP, mean ± SD (mmHg)	117.2 ± 12.59	113.70 ± 11.98	<0.001
First visit DBP, mean ± SD (mmHg)	74.24 ± 8.57	71.96 ± 8.53	<0.001
Gestational age of first visit, mean ± SD (week)	21.79 ± 7.01	21.25 ± 7.09	0.071
FBG of first visit, mean ± SD (mmol/L)	7.32 ± 2.88	6.33 ± 2.58	<0.001
Gestational age of OGTT, mean ± SD (week)	24.81 ± 4.46	25.63 ± 3.41	0.747
FBG at time of OGTT, mean ± SD (mmol/L)	6.52 ± 1.67	5.12 ± 0.98	<0.001
Frist OGTT 1 h plasma glucose, mean ± SD (mmol/L)	12.76 ± 4.14	10.75 ± 1.90	<0.001
Frist OGTT 2 h plasma glucose, mean ± SD (mmol/L)	11.28 ± 3.27	8.99 ± 1.98	<0.001
HbA1c at time of OGTT, mean ± SD (%)	6.65 ± 3.59	5.59 ± 0.83	0.000
Frequency of prior pregnancy (%)			0.728
0	376 (62.1)	473 (63.4)	
1	190 (31.4)	231 (31.0)	
2	40 (6.6)	42 (5.6)	

^*∗*^Comparison of AIT and MNT.

**Table 2:** ORs on need of AIT derived from the stepwise logistic analysis.

Variable	OR	95% CI for OR		*P*
		Lower	Upper	
Pregestational BMI	1.08	1.03	1.13	0.002
FBG at time of OGTT	2.02	1.65	2.47	<0.001
HbA1c at time of OGTT	1.90	1.46	2.47	<0.001
OGTT 2 h plasma glucose	1.16	1.07	1.27	<0.001

**Table 3:** AUC of ROC of FBG, HbA1c, OGTT 2 h plasma glucose, and BMI for need of AIT.

Variable	Area (95% CI)	*P*	Cutoff value	Sensitivity	Specificity
FBG at time of OGTT	0.80 (0.78, 0.82)	<0.001^*∗∗*^	5.69	0.673	0.797
HbA1c at time of OGTT	0.77 (0.74,0.80)	0.044^*∗*^	5.85	0.693	0.732
OGTT 2 h plasma glucose	0.73 (0.70,0.76)	<0.001^*∗*^	10.80	0.612	0.754
Pregestational BMI	0.51 (0.44,0.58)	0.804^*∗∗*^	—	—	—

^*∗*^Curve area compared with FBG at time of OGTT; ^*∗∗*^curve area compared with 0.5.
